# On the effects of testosterone on brain behavioral functions

**DOI:** 10.3389/fnins.2015.00012

**Published:** 2015-02-17

**Authors:** Peter Celec, Daniela Ostatníková, Július Hodosy

**Affiliations:** ^1^Institute of Molecular Biomedicine, Faculty of Medicine, Comenius UniversityBratislava, Slovakia; ^2^Center for Molecular Medicine, Slovak Academy of SciencesBratislava, Slovakia; ^3^Institute of Pathophysiology, Faculty of Medicine, Comenius UniversityBratislava, Slovakia; ^4^Department of Molecular Biology, Faculty of Natural Sciences, Comenius UniversityBratislava, Slovakia; ^5^Institute of Physiology, Faculty of Medicine, Comenius UniversityBratislava, Slovakia

**Keywords:** androgen, cognition, emotions, hippocampus, behavioral neuroendocrinology

## Abstract

Testosterone influences the brain via organizational and activational effects. Numerous relevant studies on rodents and a few on humans focusing on specific behavioral and cognitive parameters have been published. The results are, unfortunately, controversial and puzzling. Dosing, timing, even the application route seem to considerably affect the outcomes. In addition, the methods used for the assessment of psychometric parameters are a bit less than ideal regarding their validity and reproducibility. Metabolism of testosterone contributes to the complexity of its actions. Reduction to dihydrotestosterone by 5-alpha reductase increases the androgen activity; conversion to estradiol by aromatase converts the androgen to estrogen activity. Recently, the non-genomic effects of testosterone on behavior bypassing the nuclear receptors have attracted the interest of researchers. This review tries to summarize the current understanding of the complexity of the effects of testosterone on brain with special focus on their role in the known sex differences.

## Introduction

Despite current efforts of the European commission to combat gender issues with respect to gender equality, men and women are different in several important aspects (Cahill, 2014). These aspects include cognitive functioning and behavioral traits. Some of these may be socially induced, but scientists have showed on intact animals that other factors such as genetics and gender itself are mostly responsible forthe sex differences in behavior and cognition. Therefore, the current research strategies are calling for including both males and females in the research in order to report the possible gender differences (Ruigrok et al., 2014). Indeed, the exact mechanisms and reasons of sex differences in brain structures that mediate some of these functional dissimilarities are unknown. Genetics and endocrine factors are the most prominent biological explanations and are interconnected. Testosterone is the major male sex hormone. It is present in women, although in much lower concentrations. Testosterone has also been intensively studied in relation to sex differences and behavioral functions. This review focuses on physiology of testosterone to give the reader understanding of the mechanisms and complexity of testosterone action and then tries to summarize the studies and experiments focusing on the functional changes in anxiety, depression, spatial abilities and memory. Readers interested in sex differences and brain structures might find the needed information in the recently published focused review (Filova et al., 2013).

### Testosterone physiology

Testosterone is produced mainly in Leydig cells of testes in males, and in ovaries in females. In both, testosterone can be synthetized in the adrenal gland cortex (Burger, 2002; Dohle et al., 2003). However, in addition to the classic steroidogenic organs such as gonads, adrenals and even placenta, the active biosynthesis of steroids also occurs in the brain (Mellon et al., 2001). This synthesis can be either *de novo* from the cholesterol, or testosterone is derived from classical steroids as is deoxycorticosterone or progesterone, which enter through blood stream into nervous system. The latter one depends on the enzymatic ability of the neural region or cell. The key regulatory enzyme is Steroidogenic acute regulatory protein (StAR) (Miller and Auchus, 2011). This phosphoprotein mediates the transfer of cholesterol from the outer to the inner mitochondrial membrane, from where cholesterol can be further processed by corresponding enzymes. The StAR gene is expressed solely in the steroidogenic tissues. However, StAR mRNA expression in a rat brain was first shown by Furukawa (Furukawa et al., 1998) and confirmed in humans and mouse brains in several regions by immunohistochemistry.

The complexity of testosterone mechanism of action is underlined by its metabolism and steroid nature. The classical view suggests genomic mechanism, i.e., after translocation into cytoplasm, testosterone binds the androgen receptor, and subsequently after transportation into the nucleus it binds on the hormone response element at DNA, where it activates or silences the expression of genes and subsequent protein synthesis (Tsai and O'malley, 1994). During recent years, a new pathway for non-genomic mechanism was shown. This can include activating the membrane receptors and thus activating the second messengers, or after translocation to the cell, testosterone can either directly activate second messenger intracellular cascade, or can bind to its respective receptor and as a complex of hormone-receptor it can activate the second messenger cascade (Michels and Hoppe, 2008). Additionally, testosterone can be changed into either estradiol by aromatase or into dihydrotestosterone by reductase. The pathway depends on the enzymatic equipment of the cells.

The synthesis of sex hormones is ultimately controlled by gonadotropin-releasing hormone (GnRH), which is produced by the hypothalamus and which stimulates the pituitary gland to release luteinizing hormone and follicle stimulating hormone, where LH increases the expression of StaR protein in target cells (Ubuka et al., 2014). GnRH secretion in adulthood is pulsatile and highest during sleep with subsequent highest peaks of testosterone to be during the early morning hours (Lord et al., 2014). Nevertheless, the testosterone levels decline gradually with aging, mainly due to the attrition of Leydig cells and hypothalamic GnRH pulse generation slow down. Rapid drop can be observed in the 6th decade of life in males.(Basaria, 2013). A higher incidence of mood disorders that occurs with aging is then related to decreased testosterone and/or other androgens. However, not all studies agree with this simple explanation. Sartorius et al. showed, that there was no decline in testosterone levels in males who self-reported to be in very good health. Indeed, a subgroup of patients who were smoking and/or obese was associated with age related decline in serum androgens (Sartorius et al., 2012). Similarly, Camacho et al. reported that lifestyle factors and body weight were more important in maintaining the plasma testosterone levels than aging itself (Camacho et al., 2013).

In any respect, the causal role of testosterone deficiency and behavioral disorders including effect on cognitive abilities is still debated. Therefore, in the next chapters we will try to summarize the main experimental studies on individual behavioral traits.

## Anxiety

Our daily decision-making as well as response to stress is our everyday experience. Indeed, many factors contribute and even more factors modify the decision-making and stress reaction, with anxiety level to be one of them. Nevertheless, it has clearly been shown, and recently reviewed that women show higher anxiety in comparison to men (Mchenry et al., 2014). From all behavioral parameters, the anxiety seems to be most sensitive to testosterone. The most cited paper analyzing the effects of testosterone on anxiety in mice has shown in several experiments that testosterone—either endogenous or exogenous decreased anxiety in elevated plus maze (Aikey et al., 2002). In addition, the same study showed that this anxiolytic effect of testosterone is dose-dependent and very likely mediated by 5-alpha reductase that reduces testosterone to dihydrotestosterone. The study was conducted in male mice, but similar anxiolytic effects of single testosterone administration resulted in reduced fear of healthy women (Van Honk et al., 2005). In rats, a single testosterone injection did not reduce anxiety, however, a repeated administration had anxiolytic effects tested by the burying behavior test (Fernandez-Guasti and Martinez-Mota, 2005). A possible mechanism can include the androgen receptor, as its blockade has been shown to prevent the testosterone-induced anxiolysis. Similar results were obtained in our experiment. However, the anxiolytic effect was observed only in the light-dark box. We were not able to reproduce the anxiolytic effects of testosterone in the elevated plus maze and in the open field (Hodosy et al., 2012). On the other hand, flutamide alone had anxiolytic effects in the open field. This suggests that the association between testosterone and anxiety might not be linear. A number of experiments on gonadectomized rats from the lab of professor Frye further showed that dihydrotestosterone 3-alpha metabolites can be the mediators of testosterone anxiolytic effects (Edinger and Frye, 2004, 2005). In addition, blockade of the dihydrotestosterone transformation to 3-alpha androstanediol by a 3-alpha hydroxysteroid dehydrogenase inhibitor prevented the anxiolysis (Frye and Edinger, 2004). Age-related decline in cognitive and affective functions was associated with lower concentrations of testosterone metabolites in the hippocampus. Again, this effect blocked by administration of 3-alpha metabolites administration (Frye et al., 2010). Another mechanism of anxiolytic effect of testosterone was explained in recently published experiment, where exogenous or endogenous opioids could modulate anxiolysis (Khakpai, 2014). In this study, the gama aminobutyric acid systemthat has been proposed in the past, on the other hand, did not alleviate the anxiety level (Roohbakhsh et al., 2011). An important determinant of the postnatal association between anxiety and testosterone or its metabolites might be prenatal stress. Stress induced during gestation resulted in both, reduced testosterone and increased anxiety of the adult offspring (Walf and Frye, 2012). Taken together, the results are consistent and despite differences in the methodology it seems clear that testosterone reduces anxiety in both genders. Its higher concentrations in men might be the reason for the sex differences in anxiety. However, a very important study in rhesus monkeys showed that pharmacological castration reduced and testosterone supplementation normalized anxiety levels (Suarez-Jimenez et al., 2013). A result that is in contrast to the majority of literature of experiments in rodents. Of course, this discrepancy might be discussed with major differences in the methodology—other behavioral tests, and in the intervention—surgical vs. pharmacologic castration. But in general, experiments on monkeys are more relevant to human behavior and, thus, this study must not be overseen. Some of the animal experiments on testosterone and anxiety are summarized in Table 1.

**Table 1:** **Selected animal studies analyzing the relationship between testosterone and anxiety**.

**Species**	**Strain**	**Gender**	**Timing**	**Intervention**	**Maze**	**Mechanism**	**Outcome**	**References**
Rattus norwegicus	Wistar	Males	Young adults	Intact rats administered single injection of testosterone propionate or flutamide	Open field L/D box EPM	Flutamide as androgen receptor blocker administered	Testosterone ↑ time in L/D box by 80% vs. Control group, probably through androgen receptor; (anxiolytic effect)	Hodosy et al., 2012
Mus musculus	129:C57BL/6J	Females	Perinatal period	OVX at 28th day postnatally and capsule insertion with TP; tested on PD 67-78	Tail ST Marble burying L/D box	Organizational or activational effect of TP administration	Testosterone groups showed ↑ immobility after TP administered early postanatally; ↓ marble burying in all TP groups; no differences in L/D box; (depressive and anxiogennic effect)	Goel and Bale, 2008
Mus musculus	C57B1/6J-NHsd	Males	Young adults	Daily injections of testosterone propionate ip	Condition place preference (L/D box)	Observational/pharmacological	Testosterone did not increase L/D transitions (no effect); nandrolone showed decreased transitions by 30% at low doses; (anxiogenic effect)	Parrilla-Carrero et al., 2009
Rattus norwegicus	Long Evans	Males	Perinatal	Tfm and WT rats gonadectomized at postnatal day 1 and at postnatal day 120; further either GDX or sham with testosterone capsule implantation	Open field NOR L/D box EPM	Organizational or activational effect	Neonatal castration ↓ anxiety traits in all tests; no difference between Tfm and WT; (anxiolytic effect that is AR independent)	Zuloaga et al., 2011
Macaca mulatta	Rhesus monkey	Males	Young adults	GnRH-agonist injection to suppress testosterone secretion; then either oil or testosterone im	Modified human intruder test	Observational/pharmacological	Testosterone increased anxious behavior when compared GnRH agonist by 300%, but not vs baseline; (anxiogenic effect)	Suarez-Jimenez et al., 2013
Rattus norwegicus	Long-Evans	Males	Young adults	Gonadectomized and capsules with DHT or sham implantation	Open field EPM Defensive freezing	Observational/pharmacological; indomethacin blockade of DHT conversion to 3-alpha-diol in hippocampus	Intact and DHT replaced more active, exploratory and more time freezing than GDX; (anxiogenic effect of DHT)	Frye and Edinger, 2004
Rattus norwegicus	Long-Evans	Males	Young adults	SHAM, GDX and GDX+testosterone supplementation	Open field Social interaction Defensive burying Paw lick Emergence test EPM	Observational/pharmacological study	Testosterone ↑ time in the open arms of EPM vs GDX but not intact; (anxiolytic effect)	Frye and Seliga, 2001
Rattus norwegicus	Long-Evans	Males	Young adults	GDX + testosterone, DHT and 3-alpha diol capsules	EPM Open field Defensive burying Inhibitory avoidance	Observational/pharmacological study	Systemic and intrahippocampal testosterone decreased anxiety in open field by 250% and EPM by 200% and decreased fear behavior by 28%; The effect of testosterone was not higher than in DHT or 3-alpha diol administration	Edinger and Frye, 2004
Rattus norwegicus	Wistar	Females	Young adults	Testosterone Picrotoxin Formestane Tamoxifen	Defensive burying Open field	Pharmacological intervention, and androgen/estrogen receptor blockade	Testosterone reduced cumulative burying time vs oil by 35%; no difference in open field and burying latency (anxiolytic effect of testosterone); effect mediated through androgen metabolites, not aromatization to estradiol	Gutierrez-Garcia et al., 2009

*Tfm, testicular feminization mutation; WT, wild type; AR, androgen receptor; PD, postnatal day; OVX, ovariectomy; GDX, gonadectomy; TP, testosterone propionate; DHT, dihydrotestosterone; EPM, elevated plus maze; NOR, novel object recognition test; L/D box, light/dark box; Tail ST, tail suspension test; ip, intraperitoneally; sc, subcutaneous; im, intramuscular*.

## Depression

Although the depressive disorder is more prevalent in females (Bebbington, 1996) when compared to males, the prevalence of depression in males increases with age (Khera, 2013) as plasma testosterone drops. Consequently, many experiments and studies were performed to confirm the causative role of testosterone decline in depression pathogenesis. Indeed, these studies were not triggered by the lone fact of testosterone decline and sex difference in prevalence of depressive disorders. In depressive disorders with decreased libido and low testosterone, the androgen hormone replacement therapy was at least as effective as serotonin reuptake transporters (Kranz et al., 2014). Further it was investigated that testosterone can modulate serotonergic transmission, where serotonin plays a crucial role in depression development (Jovanovic et al., 2014). But it is not only the prevalence of depression that differ between sexes. In a study on opposite sex twins, it has been demonstrated that also the etiology of depression is different in men and women (Kendler and Gardner, 2014). Whether testosterone plays a major role in the sex differences in depression is unclear, but a number of studies indicate that it can affect the mood of depressive patients as well as healthy probands (Mchenry et al., 2014). Nevertheless, it is only one of many biological factors potentially responsible for the sex differences in depression. These were reviewed recently (Altemus et al., 2014). Observational studies on older men revealed that their depressive symptoms are associated with low plasma testosterone (Joshi et al., 2010). Low testosterone and depressive symptoms are both associated with the risk of falls, which are important for life expectancy in the elderly (Kurita et al., 2014). Similarly, in women testosterone concentrations are lower in depressive patients when compared to healthy controls (Kumsar et al., 2014). However, standard antidepressant treatment leads to normalization of testosterone. This suggests that the causality could be different than predicted—depression lowers testosterone. On the other hand, in both men and women, testosterone supplementation leads to improvement of depressive symptoms (Pope et al., 2003; Miller et al., 2009). However, not all interventional studies confirmed the anti-depressant effect of testosterone. At least in one published randomized controlled trial, the effects of testosterone were comparable to placebo effects (Seidman et al., 2001). Similarly, not all observational studies show a consistent picture. At least in one small study, depressive women had higher testosterone (Weber et al., 2000). When publication bias and the high intra- and inter-individual variability of testosterone are taken into account, these small negative or contradictory studies could be even more important. The meta-analyses of the published studies are also to be taken into account. In a meta-analysis of the effects of testosterone on depression, the anti-depressant effect was positive, at least in patients suffering from hypogonadism (Zarrour et al., 2009). The biology of the association between testosterone and depression has been reviewed recently (Mchenry et al., 2014). In an animal model of aging the associated depressive-like behavior correlated with lower testosterone (Egashira et al., 2010). Aged mice of both sexes benefited from testosterone supplementation. In the forced swim test the aged mice treated with testosterone or its metabolites spent less time immobile suggesting that the antidepressant effect of testosterone is mediated via several pathways including the androgen and the estrogen receptor (Frye and Walf, 2009). Another experiment on intact rats revealed that the effect of testosterone on depression is dose-dependent (Buddenberg et al., 2009). Interestingly, similar experiment on gonadectomized rats showed that the testosterone metabolite—3-alpha androstanediol, but not testosterone reverted the depression induced by gonadectomy (Frye et al., 2010). Selected animal experiments on the effects of testosterone on depression are compared in Table 2.

**Table 2:** **Selected animal studies analyzing the relationship between testosterone and depression**.

**Species**	**Strain**	**Gender**	**Timing**	**Intervention**	**Maze**	**Mechanism**	**Outcome**	**References**
Mus musculus	129:C57BL/6J	Females	Perinatal	Testosterone administered sc at 1st postnatal day; OVX at 28th postnatal day with testosterone capsule insertion	Tail ST	Organizational or activational effect	↑ immobility time in intact and all testosterone groups (depressive effect of testosterone)	Goel and Bale, 2008
Rattus norwegicus	Wistar	Males	Young adults	Testosterone application 15 min before testing in 3 doses (1, 2, 4 mg/kg)	FST	Observational	Failed to confirm main group effect of TST for immobility; 2 and 4 mg/kg groups spent less time immobile during 2nd trial (antidepressant effect)	Buddenberg et al., 2009
Mus musculus	SAMP10 SAMR1	Males	28–34 weeks	No intervention	Tail ST	Observation	SAMP10 prolongation in immobility time of tail suspension in comparison to SAMR1; SAMP10 showed lower TST levels but not DHEA; SAMR1 and SAMP10 showed significant correlation of TST and immobility; r= −0,667	Egashira et al., 2010
Mus musculus	C57/BL6	Males and Females	24 months (range 20–28)	Intact aged mice; 1 h before testing 1 mg/kg of TST, E2, DHT, or 3-alpha diol administered sc	FST	Observational	Main effect of sex and androgen for immobility; Aged male ↑ time immobile compared to other male groups; Aged female mice were less immobile than aged male mice (antidepressive effect of androgens and E2)	Frye and Walf, 2009
Rattus norwegicus	Sprague-Dawley	Young adults	Males and females	Gonadectomy in adulthood; Females masculinized by TP at PD 1	Learned helplessness Avoidance test Tested in adulthood	Observational/mechanism either TST or E2 organization/activation effect	All Males not able to learn to escape stress during training; all females learned to escape; TST and metabolites from periphery did not influence depressive behavior through organizational effect	Dalla et al., 2008
Mus musculus	C17/BL6	Males	8–10 weeks	ArKO mice and null KO	FST	Mechanism—through AR receptor?	ArKO did not differ in FST; ArKO exhibited normal levels of motor activity, anxiety and depression; CUMS had no effect	Dalla et al., 2005
Rattus norwegicus	Wistar	Males and females	Prepubertal and young adult males and females in estrus	No intervention	FST	Observation	Prepubertal rats of both sex increased immobility, adult males higher immobility than adult females; (depressive effect of testosterone)	Martinez-Mota et al., 2011
Rattus norwegicus	Wistar	Males and females	Young adults and 12–15 months adult rats	Gonadectomy in younger TST containing pellets in older animals	Anhedonic test CUMS		TST before CUMS prevented anhedonia in older rats; in young, gonadectomy did not increase vulnerability to anhedonia	Herrera-Perez et al., 2012
Rattus norwegicus	Sprague-Dawley	Males and females	Young adults	Gonadectomy, with pellet of TST or imipramine	Anehodonia test Novelty induced hypophagia	Observational	Testosterone had anxiolytic and antidepressant effect in males but not in OVX females; same effect as imipramine	Carrier and Kabbaj, 2012

*FST, forced swim test; CUMS, chronic unpredictable mild stress; Tail ST, tail suspension test; TST, testosterone; TP, testosterone propionate; E2, estradiol; DHEA, dehydroepiandrosterone; SAMP, senescent prone mice; SAMR, senescent resistant mice; ArKO, androgen receptor knock-out; PD, postnatal day; sc, subcutaneously*.

## Spatial abilities

Spatial cognitive abilities as well as general cognition and memory decline with aging together with the testosterone levels. During the productive ages and even in early adulthood, men generally outperform women in spatial abilities (Linn and Petersen, 1985). Especially, mental rotation shows a clear sex difference in favor of men. Not surprisingly, observational studies have focused on the association between testosterone and spatial abilities. Some studies have found a positive relationship between testosterone and mental rotation in men (Silverman et al., 1999). Error rate as well as the reaction time negatively correlated with testosterone (Hooven et al., 2004). However, it is not only the actual concentration of testosterone that is studied in relation to spatial performance. Prenatal testosterone and its proxy—the finger length ratio (second to fourth digit) seem to have a stronger association with figure-disembedding and targeting, as additional spatial abilities (Falter et al., 2006). In this study, mental rotation was affected only by sex. In another study, actual testosterone was not associated with spatial abilities, but prenatal testosterone correlated positively with spatial abilities in women (Kempel et al., 2005). In line with these findings is the lack of an association between actual salivary testosterone levels and mental rotation in men and women (Puts et al., 2010). However, in a large observational study analyzing spatial abilities in adult men from various age categories, low testosterone was associated with better spatial visualization (Yonker et al., 2006). In a very interesting study, it was found that in men, the pubertal concentrations of testosterone are negatively associated with mental rotation in the adulthood (Vuoksimaa et al., 2012). In the same paper, the comparison of twins is reported. The twin with higher testosterone scored worse in the mental rotation tests. The results are contradictory, but may depend on the test used for the assessment of spatial abilities. When virtual Morris water maze was used, a positive correlation between testosterone and spatial navigation was found in women, but not in men (Burkitt et al., 2007). The size of the corpus callosum seems to add complexity in the relationship between spatial abilities and testosterone (Karadi et al., 2006). This might be one of the causes for negative findings in studies where some of the determinants are missing (Kubranska et al., 2014). Another cause is likely the selection of the tested population. In gifted children, a negative correlation between salivary testosterone and spatial abilities was found (Ostatnikova et al., 1996). In Chinese men, the accuracy in mental rotation tests was comparable to Americans, but the reaction times were longer indicating that cultural differences could add to the variability of published results (Yang et al., 2007). Last but not least, genetic factors likely modulate the effect of testosterone. We have previously shown that at least in gifted boys, genetic polymorphisms influencing testosterone metabolism affect also its relationship to mental rotation (Celec et al., 2009, 2013). Especially, the CAG short tandem repeat in the exon 1 of the androgen receptor gene seems to be important for the action of testosterone and its metabolites (Nowak et al., 2014). Despite all complexity, the current picture indicates that the association between testosterone and spatial abilities is curvilinear and sex-dependent. In women higher testosterone is associated with better mental rotation, in men lower testosterone is associated with better spatial abilities. This seems to be true both for actual testosterone (Moffat and Hampson, 1996) and for prenatal testosterone (Grimshaw et al., 1995). Supplementation of testosterone in older men results in improvement of spatial abilities, but it is accompanied with changes in estradiol metabolism and it is likely that this interferes with modifications of spatial abilities (Janowsky et al., 1994). Even in rats, testosterone administration affects the strategy of the animals in spatial tasks (Spritzer et al., 2013). However, the interaction between testosterone and mental rotation tests is bidirectional. It has been shown that mental rotation testing affects testosterone, at least in women (Durdiakova et al., 2012). In Table 3, published experimental data on the effects of testosterone on spatial abilities are summarized.

**Table 3:** **Selected animal studies analyzing the relationship between testosterone and spatial abilities**.

**Species**	**Strain**	**Gender**	**Timing**	**Intervention**	**Maze**	**Mechanism**	**Outcome**	**References**
Rattus norwegicus	Sprague-Dawley	Males and females	Young adults	Gonadectomy or sham with or without testosterone capsule implantation (25 mg TST)	12-arm radial maze	Observational/pharmacological study	Intact males performed better in working memory tasks than intact females; Castration impaired working memory but not reference memory performance in males	Gibbs and Johnson, 2008
Rattus norwegicus	Wistar	Males	Young adults	Gonadectomy, TP 0.0625–1.0 mg sc or oil each day	8-radial maze, MWM	Observation	No effect of TST in 8-radial maze, and in MWM in terms of main effect of treatment	Spritzer et al., 2011
Rattus norwegicus	Wistar	Males	Young adults	CA1 injections of TST or DMSO or intact	MWM	Observation	TST increased latency times (worsened memory) by 200% to intact and 100% in DMSO group	Emamian et al., 2010
Rattus norwegicus	Wistar	Males	Intact/sham castrated/dolesce; If castrated then at PD 22, but trained at PD 28,35,45, and 60	TST or oil applied between PD 30-37	MWM	Observation	Pre-pubertal castration improved spatial ability in mid dolescence, but no effect in adults	Moradpour et al., 2013
Rattus norwegicus	Sprague-Dawley	Males	Young adults	GDX with TST, DHT, E2 or oil capsule sc	Object location memory	Observation	GDX impaired spatial memory; TST, E2 and DHT reversed effect	Mcconnell et al., 2012

*GDX, gonadectomy; TST, testosterone; DHT, dihydrotestoterone; E2, estradiol; TP, testosterone propionate; MWM, Morris water maze; PD, postnatal day; sc, subcutaneously*.

## Memory

Women have better verbal memory, while men have an advantage in visual-spatial memory (Lewin et al., 2001). Especially, the difference in spatial memory has been studied in detail (Shah et al., 2013). In a meta-analysis of animal experiments using radial and water mazes, it has been confirmed that males outperform females in spatial memory tasks (Jonasson, 2005). The positive effect of testosterone on memory was, however, well documented in both sexes. Numerous clinical studies in postmenopausal women and men in the andropause showed improvements of learning and memory after testosterone supplementation. Even a short 6-week testosterone treatment resulted in improved spatial and verbal memory of older men (Cherrier et al., 2001). Testosterone has even showed a positive effect on spatial and verbal memory in Alzheimer disease patients (Cherrier et al., 2005). In young women, a single dose of testosterone improved spatial memory (Postma et al., 2000). However, the mechanism of action is unclear, as testosterone is now rather considered as a precursor than as a final hormone. In contrast to some animal experiments, observational studies in elderly men showed that lower testosterone, especially its free fraction was associated with worse visual-spatial memory (Moffat et al., 2002). This might be related to the tasks used, as the testosterone levels in men are related to the learning strategies, especially for spatial memory (Choi and Silverman, 2002). The results are, however, inconsistent. In a study analyzing the effects of a single testosterone injection on elderly men the treatment caused a worsening of verbal memory (Wolf et al., 2000). Similarly, biweekly injections of testosterone during 90 days resulted in memory decline (Maki et al., 2007). In addition, patients with prostate cancer that need hormonal castration via androgen deprivation therapy had worse verbal memory than heathy controls. Interestingly, estradiol—the estrogen metabolite of testosterone reversed the negative effects of androgen deprivation (Beer et al., 2006). Similar findings were found in elderly men and women where estradiol slowed down the age-related memory decline (Carlson and Sherwin, 2000). It seems that the effect of testosterone is dose-dependent and could be curvilinear even within sexes. At least in men, it has been demonstrated that moderate dosing resulted in improved memory, but not low and very high increases of testosterone (Cherrier et al., 2007). Similar results were found in adult male rats where only moderate testosterone doses resulted in spatial memory improvements (Spritzer et al., 2011). A relatively high dose of testosterone had no effect on memory or other analyzed behavioral measures in postmenopausal women in a well-designed and large study (Kocoska-Maras et al., 2011). In another study, women with surgically induced menopause received testosterone or placebo in addition to estrogen supplementation. Testosterone in this case worsened the verbal memory (Moller et al., 2010). A smaller, but longer study on postmenopausal women showed the complete opposite—improvement of verbal memory after testosterone treatment (Davison et al., 2011). Similarly to other behavioral measures memory will be influenced also by prenatal concentrations of testosterone (Bull et al., 2010). This effect might be mediated by the organizational effect of testosterone on brain structures such as amygdala or hippocampus (Ackermann et al., 2012). It has been shown that prenatal and neonatal testosterone affects stress coping and the effects of stress on learning abilities, at least in rodents (Shors and Miesegaes, 2002). Of course, genetic factors might also play a role. At least in one study the APOE genotype interacts with testosterone regarding the influence on age-related cognitive decline (Panizzon et al., 2014). More such studies can be expected in the near future.

Animal experiments help us to uncover the molecular and physiological mechanisms behind the phenotype correlations seen in human studies. The organizational effect of testosterone on the hippocampus, the major memory structure in the brain has been described a long time ago in rats using various mazes (Roof and Havens, 1992; Roof, 1993). In birds, evidence exists for a low testosterone period needed during the development of brain functions such as vocal memory (Korsia and Bottjer, 1991). In aged rats, an important experiment showed that the positive effect was found only when testosterone was administered. The testosterone metabolite, dihydrotestosterone, which cannot be metabolized to estradiol did not showed this effect (Bimonte-Nelson et al., 2003). This indicates that the effect of testosterone on memory is mediated by estradiol and the effect of aromatase which converts testosterone to estradiol. However, in male deer mice it has been shown that aging but not testosterone affects memory (Perrot-Sinal et al., 1998). Testosterone might rather increase synaptic plasticity as shown in rats (Schulz and Korz, 2010). Increased plasticity, however, only enables improved memory. But whether the potential is used depends on other factors including environment and timing and form of learning. Another advantage of animal experiments is the possibility to surgically localize the administration of testosterone into specific brain structures, which is ethically not possible in humans. Such studies showed that in adult male rats administration of any dose of testosterone or the androgen receptor blocker flutamide resulted in worsening of spatial memory (Naghdi et al., 2001). Similar injections of flutamide into amygdala had no effect on spatial memory, but testosterone negatively affected spatial memory and learning (Naghdi et al., 2003). When histological analyses were conducted, it was found that the intrahippocampal injections of testosterone led to an increase in the number of astrocytes in the target area (Emamian et al., 2010). Co-administration of a protein kinase AII inhibitor resulted in a synergistic negative effect on spatial memory (Khorshidahmad et al., 2012). Interestingly, the injection of anastrozole—an aromatase inhibitor resulted in improvement of spatial learning and memory tested in the Morris water maze (Moradpour et al., 2006). This further confirms that the negative effect of testosterone on memory is localized to hippocampus and is mediated by estradiol. When dihydrotestosterone—the androgen metabolite of testosterone was injected into the CA1 region of the hippocampus, spatial memory was improved (Babanejad et al., 2012). Testosterone has very likely an important role in the physiology of brain functions, but it might also be useful in some pathologies. In castrated rats, testosterone was able to reverse the ethanol-induced memory deficit (Khalil et al., 2005). In diabetic rats, the memory impairment was partially reversed by testosterone administration as well (Nayebi et al., 2014). An experiment in mice contributed to the growing list of confounding variables with the length of the photoperiod. Castration and supplementation with testosterone had no effect when the photoperiod was long (16 h of light per day). On the contrary, in mice housed with a short photoperiod (8 h of light per day), the effects on spatial memory were clearly seen (Pyter et al., 2006). A selection of the numerous animal experiments focusing on testosterone and memory are presented in Table 4.

**Table 4:** **Selected animal studies analyzing the relationship between testosterone and memory**.

**Main objective**	**Method**	**Result**	**References**
Whether long term TST restoration improves vasopressin innervations and spatial learning memory	Three groups of male rats by age (young, middle aged and senescent) treated with TST or sham in MWM	TST treatment did not improve spatial learning or retention of spatial information. Aged rats performed worse than young	Goudsmit et al., 1990
If testosterone improves spatial abilities in adulthood, when administered neonatally	Testosterone propionate applied to neonatal rats; males and females, tested in adulthood	TST increased performance in control group males outperformed females, in TST group the pattern was reversed	Roof, 1993
Spatial learning and circulatory levels of testosterone in plasma	Males and females of Meadow voles according to TST and E levels underwent MWM	Male superiority was evident only with high estradiol female group, no difference between high and low TST groups	Galea et al., 1995
Whether TST treatment neonatally affects spatial leasing in adulthood in gonadectomized rats with frontal cortical lesion	Neonatally gonadectomized rats (females and males) with or without testosterone treatment underwent MWM in adulthood	Lesions at day 7 did not impair spatial learning but gonadectomy or testosterone propionate impaired the learning	Kolb and Stewart, 1995
If chronic administration of anabolic-androgenic steroids improve spatial cognition	Three groups of males supplemented with nandrolone, oil and steroid cocktail for 12 weeks, then MWM	No differences in spatial tasks in any of the treated groups	Clark et al., 1995
Investigate the effect of reproductive status on spatial learning in several reproductive stages	Meadow voles and deer mice tested in MWM either in breeding or non-breeding stage in adulthood or as juvenile	Better performance of males when females in estrus, otherwise no difference; High-E females performed worse than low-E females or males. No difference until adulthood	Galea et al., 1996
How testosterone supplementation influences spatial learning after frontal lesions in both sexes	Eight groups in experiment, females (treated with testosterone or vehicle) and males (gonadectomized or sham), all groups moreover either with frontal cortex lesion or sham	No difference of sex or hormonal manipulation, but males with lesion performed better than females with lesion	Forgie and Kolb, 1998
Spatial learning in male deer mice in relation to age	Four groups of deer mice divided by age performed in MWM. Mice were divided according to breeding state	Young and young breeding mice performed better (higher TST) than old and even young non-breeding (lower TST) mice	Perrot-Sinal et al., 1998
If prenatal androgen and estrogen affects adult spatial learning	TST and DHT females, EB females and flutamide males with prenatally (day 16) treatment were tested in adulthood in MWM	TST and EB sex differences observed in MWM as a prenatal component	Isgor and Sengelaub, 1998
If there is difference in spatial memory in females through oestrus	Male and female rats tested in MWM during several estrus cycles	No overall sex difference I retention spatial memory, females latency in estrus was longer	Healy et al., 1999
If androgen exposure impairs cognitive functions in SHR	Implantation of TST neonatally, and tested in MWM on 45th day	Androgen impaired spatial memory in SHR	King et al., 2000
Testosterone and flutamide effect on spatial performance	Intrahippocampal administration of TST or flutamide 30 min prior testing in MWM	Increased latencies in both treated groups, dose dependent	Naghdi et al., 2001
Role of sex steroids in apoE4 induced cognitive impairment	Mice expressing human apoE4 or E3 treated with testosterone and tested in MWM	Treatment improved memory deficits in apoE4 females	Raber et al., 2002
Developmental androgen sensitivity of CA3 area and spatial performance	Neonatally TST or ovariectomized females and TST castrated and TST treated or not performed in MWM during adulthood	High androgen groups did better than low androgen groups	Isgor and Sengelaub, 2003
If testosterone and flutamide in amygdale affect the spatial abilities	Testosterone or flutamide administered into amygdale 30 min prior testing in MWM	Testosterone dose dependent increase in latency times, no effect of flutamide	Naghdi et al., 2003
If testosterone improves cognition in older rats	Young and old TST or DHT treated rats underwent water radial maze	TST (but not DHT) improved spatial memory in older rats	Bimonte-Nelson et al., 2003
If testosterone improves spatial cognition in female rats	TST, DHT, Estradiol or control ovariectomized female rats tested after 48 h in MWM	Estradiol impaired spatial acquisition. TST and DHT without effect	Frick et al., 2004
Compare wild-types and testicular feminization mutation rats	Tfm and control male rats and heterozygote females performed in water maze	Males control outperformed females; Tfm showed intermediate performance	Jones and Watson, 2005
Evaluate effects of testosterone and ethanol on spatial cognition	Male rats castrates with ethanol, testosterone or both performed in water maze	Ethanol induced deficits in spatial cognition; testosterone reversed this effect	Khalil et al., 2005
Role of testicular hormones on spatial abilities	Castrated and intact males performed in MWM and delayed-matching-to-place MWM	Castration impaired working memory retention, reversed by exogenous testosterone	Sandstrom et al., 2006
If photoperiod affects spatial learning through testosterone reduction	Mice either in 16 or 8 h daylight for 14 weeks performed in MWM after castration/sham/castration+ testosterone	Castrated with testosterone short day mice performed better to other short day mice, in long day no differences	Pyter et al., 2006
Evaluate effect of TST, estrogen and anastrazol on spatial abilities	CA1 cannulation of adult male rats with various dosages of testosterone, estradiol, anastrazol or DMSO	TST and estradiol impaired spatial learning, anastrazol improved it	Moradpour et al., 2006
If castration of males affects the spatial memory	Castrated and sham male rats performed in MWM	No differences between groups	Spritzer et al., 2008
Spatial learning and TST	Castrated and intact male rats, rats cannulated into right or left hippocampus castrated or not did spatial task in MWM	Castration did not affect learning	Mohaddes et al., 2009
Investigate effects of TST metabolites on spatial performance	Male rats subjected to orchiectomy and capsule with TST metabolites implanted did spatial tasks in MWM	3-alpha and 3-beta-diols enhanced spatial cognition	Osborne et al., 2009
Enhancement of aged female rats by androgen supplementation	Old mice with implanted TST or DHT or empty capsules performed in MWM after 6 weeks	TST improved spatial cognition, DHT did not	Benice and Raber, 2009

*TST, testosterone; DHT, dihydrotestoterone; EB, estradiol benzoate; Tfm, testicular feminization mutation; DMSO, dimethytlsulfoxide; MWM, Morris water maze*.

## Functional magnetic resonance imaging in humans

Functional magnetic resonance imaging (fMRI) is a neuroimaging procedure that uses MRI technology for measuring the brain activity. The principle lies in detection of associated changes in blood flow and is useful in mapping the brain functional areas (Hofer et al., 2013). Several studies were performed using human volunteers for spatial tasks, memory as well as mood disorders/traits.

As for spatial tasks and mental rotation, the fMRI data are valuably consistent. In line with the previous studies, the males outperformed females in spatial tasks. Additionally, the fMRI showed stronger activation of left inferior parietal lobe in males compared to females. Also, the testosterone levels correlated with activation levels during mental rotation task in males. In females, the early follicular and midluteal phases were associated with better outcome and higher estradiol concentrations (Schoning et al., 2007). Likewise, a study of van Hemmen et al. confirmed previously reported sex differences in neural activation during mental rotation. Moreover, participants with complete androgen insensitivity syndrome presented with female-like neural activation pattern in the parietal lobe, indicating that gonadal hormone exposure rather than genetic sex itself plays role in brain functions (Van Hemmen et al., 2014). The menstrual cycle and thus the involvement of sex hormones, including testosterone, in spatial abilities was further confirmed by Pletzer et al. In their study, error rates linked with deactivation of inferior parietal lobes and prefrontal lobes were higher during luteal phase for verbal tasks, while in the follicular phase, spatial abilities in females were confirmed (Pletzer et al., 2011).

## Issues

### Psychometric tests

The analysis of behavior is not as straightforward as biochemical and molecular methods. Several alternatives exist for testing any brain function. However, the tests are variable and it is only a consensus, which can or should be used. The same applies to mazes used for the assessment of animal behavior. Even for the widely used Morris water maze several alternatives exist and numerous different parameters are used in the particular studies. An experiment showed that testosterone does not affect some of the measures analyzed in the water maze, but does affect other measures such as spatial working memory retention (Sandstrom et al., 2006).

### Populations

One of the major factors that might explain the differences between the results of various studies is the variability of the examined populations. As mentioned above, the cultural differences, sex and age have all been shown to impact the physiological effects of testosterone. In animal experiments, chosen species and the particular strain is also of importance. Looking at the studies in non-human primates in contrast to the majority of rodent studies the results are mostly negative. For example, testosterone manipulations in rhesus monkeys did not alter their working and reference memory, although emotional processing was affected. Indeed, the treatment for testosterone might have not last long enough to affect the cognition (Kelly et al., 2014). Other possible explanations might be due to low number of animals included, but also to physiological differences including body size and the concluding testosterone kinetics (King et al., 2012). Specific behavioral tests might also be responsible for the differences observed (Lacreuse et al., 2012).

### Testosterone measurement

There are several possibilities as to what kind of biological samples should be used for the testosterone measurement. Plasma, saliva, urine are available and all have some strengths and weaknesses. The simple scheme of free—bioactive fraction of testosterone that should be assessed using salivary testosterone or plasma albumin and sex hormone binding globulin is not correct. Bound testosterone has its effects on target tissues and it is not clear which of the potential biological liquids is robust against technical and biological variability. One of the exotic possibilities is measurement of testosterone in the hair. The concentration in the hair might, however, be relevant as it integrates all the intra-individual variability of testosterone (Dettenborn et al., 2013).

### Timing

Testosterone undergoes several biorhythms. In some studies, even the best-known circadian rhythm is not taken into account. Implants that slowly release testosterone totally ignore daily variations that occur physiologically. Other rhythms such as infradian cycles are completely forgotten when experiments are designed. But beyond cyclic variations, testosterone undergoes chaotic temporary changes that are usually described as noise. Although such research is lacking, it might be that it has some physiological role similarly to heart rhythm variability. In addition, the timing of behavioral analyses is of importance. While within 30 min after administration, non-genomic effects are important, later genomic effects are expected to be the major mediator. But this does not have to be true. Even later, the non-genomic effects are active in parallel with the gene expression changes. Only the study of the particular effects is more and more complicated, especially due to the complex kinetics of testosterone and the complex abilities being tested as proved in a focused experiment (Hawley et al., 2013). Additionally, physiological and also behavioral functions are exerted on a rhythmic basis. Timing the behavioral tests for light phase, while rodents usually are active during night can represent a major problem in animal behavior testing. Moreover, the central circadian clock is located in the suprachiasmatic nucleus of the hypothalamus and it receives signals directly from photoreceptors. GABA is thought to play a major role in coordinating the synchronized firing of suprachiasmatic neurons (Urbanski, 2011). However, steroid hormones may also exert their nongenomic function through GABA receptors. Disrupting the GABAergic system by untimed testosterone application, may be one other reason for controversy results in behavioral analysis. Alternatively, aging is strongly related to decline of circulating sex hormones, disrupting thus also circadian rhythms and leading to impaired sleep or cognitive functioning (Urbanski et al., 2014). Restoring natural circulating hormone pattern in older but also in younger animals could possibly lead also to more comprehensive results of sex hormones and behavior studies.

### Administration route

In most studies, testosterone is injected via i.p. or i.m. injections, but there are indices that to study the effects of testosterone on brain functions, the steroid has to be injected directly into the target brain structure. At least in one experiment directly comparing peripheral administration and intrahippocampal injections of testosterone it was shown that the peripheral route had no effect on learning and memory while central injections were effective (Harooni et al., 2008).

### Type of testosterone used

Testosterone in the experiments is sometimes used as butyrate, decanoate, undecanoate etc. These pharmacological forms have, however, variable kinetics and might therefore have also variable effects, especially in the brain, where the kinetics is of special importance (Filova et al., 2012). Dosing of testosterone seems to be of enormous importance. It varies between the experiments widely and should always be taken into account when evaluating the results. In experiments, moderate, but not very low or very high doses of testosterone had some effect on behavioral measures such as memory (Spritzer et al., 2011).

The effect of testosterone is influenced by several factors, but only some of them are known. These include genetic polymorphisms related to testosterone metabolism or other pathways related to cognitive functioning (Panizzon et al., 2014). Next generation sequencing and lower prices of genotyping will enable detailed studies focusing on the genetic factors and especially on the complex interactions between genetic, endocrine and other environmental factors.

### Metabolism

Testosterone is currently seen more as a precursor of hormones. In most target tissues, testosterone is converted into metabolites such as dihydrotestosterone—a more potent androgen receptor ligand. The enzyme aromatase, on the other hand, can metabolize testosterone into estradiol—a ligand of the estrogen receptors. Further metabolites are being added to the list. But in general, it is of importance to recognize the role of the target tissue that can convert testosterone to inducers of very different signaling pathways. Without genetic or pharmacologic manipulation it is not possible to distinguish the effects when testosterone itself is administered.

### Non-genomic effects

The metabolism of testosterone makes studying the physiology of testosterone effects on the brain difficult. But the response of target tissues are similarly complex. Testosterone can be recognized by the androgen receptor inducing genomic effects—changes in gene expression. But the same testosterone can induce other signaling pathways that do not require changes in the use of the genomic information. These effects are called non-genomic and are studied for all steroid hormones. When testosterone is injected into the hippocampus together with a protein synthesis inhibitor that prevents genomic effects, spatial memory is improved in male rats (Naghdi et al., 2005). This points toward the possibility that non-genomic effects can be opposite to the genomic effects. But it also shows that doing such experiments and interpreting their results is difficult. Inhibition of protein synthesis is of course not specific. An alternative is to analyze the behavior rapidly after testosterone injection, as it take roughly 30 min to induce gene expression changes. But the kinetics of testosterone *in vivo* complicates the interpretation. Another option is the co-administration of androgen receptor and estrogen receptor blockers.

## Conclusion and future outlook

While fMRI results bring interesting data and knowledge on behavioral traits and spatial abilities in relation to testosterone levels and sex differences, the result obtained can show only association or correlation but not causal relationship of testosterone effect on behavior. Nevertheless, also according to the numerous published studies and animal experiments, testosterone seems to affect brain functions. The high number of relevant publications also indicates that it is a hot topic of interest. However, quantity is not quality and currently, despite numerous publications it is very difficult to conclude how testosterone affects cognitions and emotions. Most of the published literature agrees on the fact that testosterone is anxiolytic, anti-depressant and improves spatial abilities. But this picture is oversimplified. Many variables add to the complex interactions between testosterone and the brain. Memory, both, verbal and spatial, is a good example. Age, sex, current endocrine status, but also the timing of testosterone analysis or administration, status of the target tissues and several other factors influence the outcome of observational or interventional studies. It is, thus, clear that small studies can only describe a very small window of the whole complex physiology. Analyzing testosterone concentrations, choosing appropriate doses and pharmacological forms is difficult enough. The psychometrics behind behavioral tests in animal experiments and behind psychological tests in human studies is, nevertheless, lacking. Standardization in this area would surely improve our understanding of the neuroendocrinology of testosterone. More systematic research using the whole spectrum of available tools and looking at the various physiological aspects is needed. However, to be able to publish such research, journals should accept manuscripts based on the design and not on the results. Otherwise, the publication bias that is obvious in the so far published literature will continue to be a big issue. Many researchers in this field complain about negative results that are very difficult to publish in the relevant journals. The number of such unpublished observations and experiments is unknown. But based on our humble experience, the negative results will probably be more common than the published positive ones. And if the contradictory published findings are added, the picture gets even more confusing. Large systematic research projects with more cooperation between the most productive research teams is definitely needed.

### Conflict of interest statement

The authors declare that the research was conducted in the absence of any commercial or financial relationships that could be construed as a potential conflict of interest.
